# Front-Line Therapy for Elderly Chronic Lymphocytic Leukemia Patients: Bendamustine Plus Rituximab or Chlorambucil Plus Rituximab? Real-Life Retrospective Multicenter Study in the Lazio Region

**DOI:** 10.3389/fonc.2020.00848

**Published:** 2020-06-10

**Authors:** Francesco Autore, Idanna Innocenti, Francesco Corrente, Maria Ilaria Del Principe, Serena Rosati, Paolo Falcucci, Alberto Fresa, Esmeralda Conte, Maria Assunta Limongiello, Daniela Renzi, Laura De Padua, Alessandro Andriani, Francesco Pisani, Giuseppe Cimino, Agostino Tafuri, Marco Montanaro, Francesca Romana Mauro, Giovanni Del Poeta, Luca Laurenti

**Affiliations:** ^1^Institute of Hematology, Fondazione Policlinico Universitario Agostino Gemelli IRCCS, Rome, Italy; ^2^Hematology Unit, Department of Biomedicine and Prevention, University tor Vergata of Rome, Rome, Italy; ^3^Hematology Unit, Department of Cellular Biotechnologies and Hematology, Sapienza University, Rome, Italy; ^4^Division of Hematology, Ospedale Belcolle, Viterbo, Italy; ^5^Institute of Hematology, Università Cattolica del Sacro Cuore, Rome, Italy; ^6^Hematology Unit, Azienda Ospedaliera-Universitaria Sant'Andrea, Rome, Italy; ^7^Hematology Unit, Ospedale Santa Maria Goretti, Latina, Italy; ^8^Hematology and Stem Cell Transplant Unit, IRCCS Regina Elena National Cancer Institute, Rome, Italy; ^9^Hematology Unit, Fabrizio Spaziani Hospital, Frosinone, Italy

**Keywords:** chronic lymphocytic leukemia, bendamustine, chlorambucil, rituximab, chemoimmunotherapy

## Abstract

Previous studies investigated the efficacy and the safety of bendamustine (B) vs. chlorambucil (Chl) associated with rituximab (R) in fludarabine-ineligible patients with treated and untreated chronic lymphocytic leukemia (CLL). We conducted a retrospective multicenter study in the Lazio region to further evaluate and compare the efficacy and the toxicity of Chl-R and B-R regimen in CLL patients over the age of 65. We enrolled 192 untreated CLL patients: 111 treated with B-R and 81 with Chl-R. The overall response rates (ORR; 93.6% in B-R and 86.5% in Chl-R) were not statistically different between the two groups, such as progression-free survival (PFS), time to retreatment (TTR), and overall survival (OS). The B-R group showed a higher hematological (*p* = 0.007) and extra-hematological (*p* = 0.008) toxicity. When comparing the toxicities according to age, we noted that the extra-hematological toxicity was higher in patients over the age of 75 who were treated with B-R than those treated with Chl-R (*p* = 0.03). This retrospective study confirms the feasibility of B-R and Chl-R in elderly untreated CLL patients. Currently, patients who are over 75 and unfit are usually treated with Chl-R. This scheme allows achieving the same ORR, PFS, TTR, and OS when compared with B-R because of hematological and extra-hematological toxicities due to B, in which a greater dose reduction has been shown in comparison to Chl.

## Introduction

Chronic lymphocytic leukemia (CLL) mainly affects elderly people, with a median age at onset of 72. About three quarters of the patients with newly diagnosed CLL are 65 or older, with ~42% being older than 75 and with age-related comorbidities (1).

Age in CLL has a primary role in the prognosis and in the choice of treatment. In fact, the standard first-line treatment (fludarabine, cyclophosphamide, and rituximab—FCR) is poorly tolerated in elderly patients or in patients with comorbidities (2–5).

A suitable option in elderly patients is chlorambucil (Chl) which, as a single agent, is well-tolerated, but the response rates are modest with Chl only (31 to 72%), with only a few patients achieving complete remission (CR, 0 to 7%). To improve the treatment outcomes, combinations of Chl with monoclonal antibodies obtained better results (6, 7); a phase III CLL11 study demonstrated an improved efficacy with Chl plus rituximab (Chl-R) and Chl plus obinutuzumab vs. Chl monotherapy, with a superiority of obinutuzumab compared to rituximab in patients with comorbidities (6). The overall response rates (ORR) of Chl-R ranged in different studies from 66 to 84%, with CR between 8 and 26% and progression-free survival (PFS) from 16.3 to 34.7 months (8–10).

An alternative option is bendamustine (B), of which the results were published by the German CLL study group. Their phase 2 trial investigated the safety and the efficacy of the combination of bendamustine and rituximab (B-R) in previously untreated patients with CLL. The patients enrolled in this study were relatively young, with a median age of 64 (range: 34–78), and only 25% were over the age of 70 (11). B-R is efficacious and well-tolerated with a potentially less hematologic toxicity than the fludarabine-based regimens. Notably, PFS after 2 years was similar in the elderly patients and could therefore be an appropriate therapeutic option in patients who cannot tolerate the FCR regimen (12).

Chl and B were compared in a phase 3 trial as frontline therapy in CLL patients under the age of 75. Bendamustine induced significantly higher ORR and CR rates than Chl, with a manageable toxicity profile. It also demonstrated a significant improvement of median PFS and time to retreatment (TTR) (13, 14). The randomized open-label MABLE study, which investigated the efficacy and the safety of the two regimens, B-R and Chl-R, in fludarabine-ineligible patients with untreated CLL, confirmed that both schemes were good first-line options (15). The CR rate after 6 cycles and the median PFS were higher with B-R than with Chl-R; the ORR and the overall survival (OS) were not different. Recent data about the use of Chl-obinutuzumab (6, 16) in untreated CLL patients with comorbidities and the possible use of ibrutinib (17) in untreated CLL elderly patients (>65) who do not harbor del17p or TP53 mutation lead to further therapeutic options for CLL patients who are >65 years in age.

We conducted a retrospective multicenter study to further evaluate and compare the efficacy and the toxicity of the Chl-R regimen and the B-R regimen in CLL patients over the age of 65. The aim of our study was to establish the safety and the efficacy of the two regimens in a real-life setting and to investigate whether certain CLL patients could benefit mostly from one of the two combinations.

## Materials and Methods

The study was designed as a retrospective analysis of CLL patients treated with B-R and Chl-R at eight hematological centers in the Lazio region from 2009 to 2016.

Previously untreated elderly patients (≥65 years of age) with progressive CLL or with small lymphocytic lymphoma treated with Chl-R or B-R and with a minimum follow-up of 12 months were included in the study.

We collected the main clinical and the biological characteristics of these patients and we registered their clinical impact on ORR, CR rate, PFS, TTR, OS, and hematologic or extra-hematologic toxicities. All adverse events, treatment reductions, and hospitalizations were registered.

The treatment schedule of Chl was not the same in all the centers: the majority of patients (53/81) were treated according to the schedules previously reported by Foà et al. (9) and Laurenti et al. (8); the remaining patients (28/81) were treated as reported by Goede et al. (6). R was added to Chl from the 1st or 3rd cycle onwards and was administered on day 1 of each cycle with a dose of 375 mg/m^2^ during the first administration and 500 mg/m^2^ for the subsequent five cycles. The patients treated with B-R were assigned to receive 6 monthly courses of B-R. Bendamustine was administered with a dose of 90 mg/m^2^ on days 1 and 2. Rituximab was administered with the same schedule as that of the Chl-R group. Response assessment was performed 2 months after the completion of treatment, and its definition was based on the revised iwCLL 2008 criteria (18).

The study was approved by the Institutional Review Board of the center, and all the patients provided written informed consent. The trial was conducted according to the Helsinki Declaration, Good Clinical Practice, and applicable national regulations.

Patients who were ≥65 years of age, with CLL requiring treatment according to the iwCLL criteria (18) and with performance status ≤2 as per the Eastern Cooperative Oncology Group (ECOG), were included in the study. Transformation to aggressive B-cell malignancy (Richter syndrome), active B or C hepatitis, HIV, and active non-skin neoplasia were criteria for exclusion.

The primary endpoints included the TTR and the ORR, with the responses evaluated according to the revised iwCLL 2008 criteria, with the exception of a few patients for whom a CT scan and/or a bone marrow biopsy were not available (those patients were evaluated by ultrasound and bone marrow aspiration). The secondary endpoints were PFS, OS, and hematological and extra-hematological toxicity according to the Common Terminology Criteria for Adverse Events, version 4. We did not consider the CR rate as a primary endpoint because some patients were not available for CT scan and/or bone marrow biopsy. For the same reason and considering that the study was a real-life analysis, we preferred TTR to PFS as a primary endpoint. The responses and the outcomes were correlated with clinical and biological parameters. The ORR was calculated on the intent-to-treat population, defined by all patients who received at least one dose of the study medication. Other endpoints were the response rate in biologically defined risk groups according to the harbored mutations and time-dependent outcome variables. The OS time was calculated from the first day of treatment until death from any cause. The PFS time was defined as the interval from the first day of treatment until disease progression or death, and the TTR was defined as the interval from the end of Chl-R or B-R treatment until the initiation of a new treatment or death. The safety analysis included all patients who received at least one dose of treatment.

Non-parametric tests were carried out for comparisons, and logistic regression was performed to adjust for the effect of clinical and biological factors on the ORR. The survival curves were generated using the Kaplan–Meier method. Differences in OS, PFS, and TTR were evaluated using the log-rank test. All tests were two-sided, accepting *p* < 0.05 to indicate a statistically significant difference, and confidence intervals (CI) were calculated at a level of 95%. All analyses were performed using STATA/SE 12.0 software.

## Results

### Patients

One hundred ninety-two patients who underwent treatment between 2009 and 2016 were enrolled in the study. The analysis was performed on 111 patients treated with B-R and 81 patients treated with Chl-R. Their clinical and laboratory characteristics are summarized in Table 1.

**Table 1:** Patients' clinical and biological characteristics.

**Patients' characteristic**	**B-R 111 patients**	**Chl-R 81 patients**
Median age at treatment	69 years (range: 65–81)	75 years (range: 65–85)
Age 65–75	90 (81.0%)	46 (56.8%)
Age >75	21 (19.0%)	35 (43.2%)
Male/female	65/46	50/31
Cumulative Illness Rating Scale score >6	9 patients (8.1%)	20 patients (24.7%)
Eastern Cooperative Oncology Group (ECOG) score 0	81 (73.0%)	51 (63.0%)
ECOG score >1	30 (27.0%)	30 (37.0%)
Lymphocytes count (range)	59.5 × 10^9^/L (3.0–240.0)	64.0 × 10^9^/L (3.0–240.0)
Hemoglobin levels (range)	12.8 g/L (5.8–16.6)	12 g/L (7.7–16.6)
Platelet count (range)	164 × 10^9^/L (23–472)	142 × 10^9^/L (41–362)
Binet A	33 (29.7%)	42 (51.8%)
Binet B	67 (60.4%)	33 (40.7%)
Binet C	11 (9.9%)	6 (7.4%)
Fluorescence *in situ* hybridization analysis	88/111 patients	75/81 patients
Normal karyotype	35 (39.8%)	20 (26.7%)
13q deletion	25 (28.4%)	33 (44.0%)
+12	15 (17.0%)	15 (20.0%)
11q deletion	9 (10.2%)	7 (9.3%)
17p deletion	4 (4.5%)	-
Immunoglobulin heavy chain gene	61/111	51/81
Unmutated	42 (68.9%)	29 (56.9%)
Mutated	19 (31.1%)	22 (43.1%)
Subgroup	55/111	52/81
Low-risk group	18 (32.7%)	20 (38.5%)
Intermediate-risk group	37 (67.3%)	32 (61.5%)
B and Chl dose/patient (range)	1,680 mg (200–2,700)	600 mg (210–980)
Median dose for cycle (range)	300 mg (120–450)	90 mg (60–130)
R dose/patient (range)	4,600 mg (1,270–7,750)	3,900 mg (600–7,350)
Median dose for cycle (range)	775 mg (600–1,000)	666 mg (350–1,000)

In the B-R group, the median number of B cycles was six (range: one to six) and the median dose of B administered during the treatment was 1,680 mg per patient (median dose: 300 mg for each cycle); the median number of R cycles was six (range: one to six) and the median dose of R was 4,600 mg per patient (median dose: 775 mg for each cycle). All patients started the B treatment at the standard dose of 90 mg/m^2^, except for 19 patients (17%) in which the baseline dose was 70 mg/m^2^.

In the Chl-R group, the median number of Chl cycles was six (range: three to 10) and the median dose of Chl administered during the treatment was 600 mg per patient (median dose: 90 mg for each cycle); the median number of R cycles was six (range: one to eight) and the median dose of R was 3,900 mg per patient (median dose: 666 mg for each cycle).

### Efficacy

A significantly higher CR rate was shown in the B-R arm compared to the Chl-R group (54.9 vs. 30.9%, *p* = 0.001). Nevertheless, the difference in terms of ORR between the two groups (93.6% in B-R and 86.5% in Chl-R) was not statistically relevant, due to a higher partial response rate in the Chl-R group compared to the B-R group (55.6 vs. 38.7%, *p* = 0.021).

After a median observation time of 72 (12–241) and 73 (12–210) months, respectively, in the B-R group 45/111 patients progressed (40.5%); so, the median PFS was not reached. In the Chl-R group, 45/81 patients progressed (55.6%) with a median PFS of 37 months (CI 95%, 30–39).

In the B-R arm, 38/111 patients (34.2%) required retreatment, with a median TTR of 53 months (CI 95%, 43–63); in the Chl-R arm, 39/81 patients (48.2%) required retreatment, with a median TTR of 46 months (CI 95%, 36–58). The most frequently used regimens at CLL progression were ibrutinib (29%), retreatment with Chl+/-R (31%), B-R (8%), R-CHOP (6%), and FCR (4%).

In the B-R group, 10/111 patients (9.0%) died at a median time of 31 months; 95 and 83% of the patients were alive after 3 and 5 years, respectively. The CLL-related mortality was 6.4% (seven of 111). The remaining three patients died of secondary neoplasia. In the Chl-R group, 18/81 (22.2%) died at a median time of 36 months; 87 and 78% of patients were alive after 3 and 5 years, respectively. The CLL-related mortality was 14.8% (12/81). The remaining six patients died of causes other than progressive disease (PD; two of myocardial infarction, two of infection, and two of secondary neoplasia). No statistical differences were observed between the two treatments in terms of PFS, TTR, and OS.

Among all the investigated clinical and biological characteristics [age, gender, Cumulative Illness Rating Scale (CIRS), ECOG, lymphocytes, Binet stage, fluorescence *in situ* hybridization (FISH), and immunoglobulin heavy chain gene (IGHV)], age was the only significant variable for ORR in univariate analysis in the B-R group. In the Chl-R arm, age, CIRS, and ECOG were significant in the univariate analysis, whereas age and CIRS were significant in the multivariate analysis.

When dividing patients according to age, in elderly patients (65–75 years) vs. very elderly patients (>75 years), we noted more very elderly patients in the Chl-R group (35/81 in Chl-R vs. 21/111 in B-R, *p* = 0.009). In each subgroup, we noted a worse response rate for the very elderly patients: progressions and stable diseases were higher in the very elderly patients (>75 years) rather than in the elderly population (65–75 years).

The survival curves did not differ in the age subgroups of B-R treatment, showing similar results in terms of PFS, TTR, and OS; in the Chl-R combination, the CLL patients with age >75 years showed a worse OS rather than the elderly patients(*p* = 0.037).

For a further subgroup analysis, the patients were classified according to the combination of cytogenetics FISH analysis and IGHV mutational status. We therefore focused on the intermediate-risk group (IR), including patients with 11q and/or unmutated IGHV, and the low-risk group (LR), including patients without 11q but with mutated IGHV. We excluded four patients who carried del17p, treated with B-R before the ibrutinib era, because of their poor prognosis (high-risk group). A subgroup analysis of the IR (37 patients in the B-R group and 32 patients in the Chl-R group) and the LR (18 patients in the B-R group and 20 patients in the Chl-R group) showed that no differences were found in the outcome curves for PFS, TTR, and OS between the B-R and the Chl-R groups.

### Safety

The B-R group showed a higher hematological toxicity compared to the Chl-R arm [53/111 patients (48%) vs. 23/81 patients (28%), *p* = 0.007]. Grades III and IV hematological toxicity was observed in 40/111 patients (36%) in the B-R group and in 18/81 patients (22%) in the Chl-R group (*p* = 0.039). Neutropenic events in the B-R group were reported in 45/111 patients (41%), of whom 32/111 patients (29%) were of grades III and IV. In the Chl-R group, 15/81 patients (19%) presented with neutropenia (*p* = 0.001) and 13/81 cases (16%) were grades III and IV (*p* = 0.039). Other hematological toxicities such as anemia and thrombocytopenia were registered in five (4%) and three (3%) of 111 patients in the B-R group and five (6%) and three (4%) of 81 patients in the Chl-R arm, respectively (*p* = ns). Granulocyte colony-stimulating factor was used in 19 (23%) and seven (6%) patients with grade IV neutropenia who treated with the B-R and the Chl-R regimen, respectively (*p* = ns).

The B-R arm also presented a significantly higher extra-hematological toxicity than the Chl-R group [51/111 patients (46%) vs. 22/81 patients (27%), *p* = 0.008]. In the B-R group, 27/111 patients (24%) experienced infective complications (14 pulmonary infections and 13 gastrointestinal infections) and 18/111 patients (16%) had mild cutaneous reactions during B infusion. Six out of 111 patients (5%) showed mild (grades I and II) infusion-related reactions during the first administration of rituximab.

In the Chl-R arm, nine (11%) of 81 patients presented infective complications: five pulmonary infections and four gastrointestinal infections. Ten patients (12%) experienced mild infusion-related reactions and one patient experienced a grade III reaction during the first rituximab administration; three patients showed cutaneous reactions.

When comparing the toxicities according to age, we noted that extra-hematological toxicity was higher in the very elderly patients (>75 years) treated with B-R than in the elderly patients treated with Chl-R (*p* = 0.03). No statistical significance was found upon analyzing hematological toxicity.

Bendamustine and chlorambucil reductions were recorded in 38/111 cases (34%) and in 15/81 (19%) patients, respectively. Dose reductions and toxicities were not related to age.

Thirteen patients (14%) and seven patients (6%) were hospitalized, respectively, in the B-R arm and in the Chl-R arm, with no statistical difference between the two groups. The most important causes of hospitalization were pulmonary infections, autoimmune hemolytic anemia (AIHA), and fever of unknown origin. Five patients discontinued the treatment: three patients in the B-R arm (one AIHA, one cutaneous epidermolysis, and one PD) and two in the Chl-R arm (one AIHA and one larynx carcinoma).

During follow-up, 10 patients died in the B-R arm of PD (five patients), other neoplasia (three patients), and infective complications (two patients) and 18 patients died in the Chl-R arm of PD (nine patients), heart disease (four patients), infective complications (three patients), or other neoplasia (two patients).

## Discussion

In this paper, we retrospectively evaluated two R-based regimens, specifically the Chl-R and the B-R, in elderly (>65 years of age) untreated CLL, aiming to understand whether these regimens differ in the real-life for clinical characteristics, efficacy, and toxicity. In the era of the new targeted drugs, comparing Chl-R to B-R, even if they are considered out-of-date therapies according to the most recent update (19), could be helpful in those centers where the new drugs are still not available for logistic or economic reasons. Moreover, the guidelines suggest to treat IgVH-mutated patients aged 65–70 years old with chemoimmunotherapy (CIT) (B-R of Chl-antiCD20); the use of ibrutinib is suggested for patients unsuitable for CIT. Analyzing our setting, we confirmed that both treatments can result in a similar response rate as for ORR, PFS, TTR, and OS. In particular, an excellent ORR was observed in both groups, confirming the role of Chl and B in association with R in elderly patients with CLL. Our data were similar to the MABLE first-line treatment experience. As far as Chl is concerned, even if two different schedules of administration were used, MABLE and our analysis reported the administration of a median Chl cumulative dose of 720 and 600 mg, respectively, higher than that reported in the CLL-11 study (366–400 mg). This difference may explain the better ORR observed in the MABLE and our study compared to the CLL-11 study (Table 2).

**Table 2:** Comparison of response rates and survival outcomes between our cohort of patients and those of other previously published studies.

		**Lazio study B-R/Chl-R**	**Italy study^**20**^ (B-R)**	**CLL-10^**12**^ (B-R arm)**	**MABLE^**15**^ (B-R/Chl-R)**	**GIMEMA^**8**^ (Chl-R)**	**CLL-11^**16, 21**^ (Chl-R)**
Overall response rates	B-R	93.6%	88.6%	96%	91%	na	na
	Chl-R	86.5%	na	na	86%	87.1%	65.7%
Progression-free survival (months)	B-R	46	35	48.5	39.6	na	na
	Chl-R	37	na	na	29.9	43.7	15.4
Time to retreatment (months)	B-R	53	48	ne	ne	na	na
	Chl-R	46	na	na	ne	72.3	32.7
Overall survival	B-R	−95% at 3 years −83% at 5 years	55 months 89.6% at 2 years	92% at 3 years	43.8 months	na	na
	Chl-R	−87% at 3 years −78% at 5 years	na	na	nr	86.1% at 3 years 81.2% at 5 years	nr

*na, not applicable; ne, not evaluable; nr, not reached*.

Alternatively, our better CR rate, even if it is considered as a secondary end-point, in comparison to CLL-11 and MABLE could be explained by the fact that 40 and 47% of our patients lacked CT scan and bone marrow biopsy for therapeutic response evaluation (6, 15). In fact, our CR rate is similar to that reported in a previous GIMEMA study in which CT scan and bone marrow biopsy were also not routinely used (8). No differences in terms of PFS, TTR, and OS were reported among the Chl-R and the B-R groups in our experience.

Our median PFS, not reached and 37 months after B-R and Chl-R, respectively, were slightly better than the MABLE experience (39.6 and 29.9 months, respectively). For the B-R treatment, we observed a PFS rate similar to that reported in the CLL10 study (Table 2); when comparing our patients (8% of our patients were unfit) to CLL10 fit patients over 65 years of age, the median PFS was 46 and 48.5 months, respectively (12). Also, the PFS for the Lazio Chl-R group was similar to that of the GIMEMA study with a median PFS of 37 and 43.7 months, respectively (8). TTR is comparable only with the GIMEMA experience in which it was the primary endpoint of the study. In our Chl-R arm, 48% of the patients were retreated at the median time of 46 months; the GIMEMA study reported a median TTR of 72 months. The shorter TTR in our study could be explained by the older median age of the patients (75 vs. 72 years) and, consequently, by the high rate of death registered during follow-up (10 out of 81 patients in the current study vs. six out of 103 patients in the GIMEMA experience).

In the B-R arm, retreatment occurred in 34% of patients, resulting in a median TTR of 53 months. These data are better than those published on the Italian experience with elderly patients treated with B-R (median TTR: 48 months) (20). The differences between the two groups (current study and Italian analysis) that could explain the better TTR in our study were age (69 vs. 72 years), comorbidities (9 vs. 11.4%), and del17p (4 vs. 5.6%), respectively. The Lazio study confirmed the MABLE data about the absence of differences in terms of median OS between the B-R and the Chl-R regimens. Previous phase II studies of R-B and R-Chl (9–11), even if they were a single arm, did not show any statistical differences as well. On grouping patients according to age (elderly vs. very elderly patients), there were more very elderly patients in the Chl-R group (35/81 in Chl-R vs. 21/111 in B-R, *p* = 0.009), which is likely because the clinicians' choice is related to age, with the predilection of Chl-R as the first choice of treatment in the very elderly patients. Comparing the elderly vs. the very elderly CLL patients treated with Chl-R only, OS was significantly lower in the CLL patients of age >75 years (*p* = 0.037). By contrast, in the B-R arm, very older age did not negatively affect the OS rate. Similarly, we did not observe statistically significant differences among age and the other prognostic factors analyzed as for the PFS and the TTR rates. This is probably related to a limitation due to the nature of the study itself and to the fact that, in the cohorts, only half of the patients had biological prognostic features reported at the beginning of the therapy. Another limitation is the difficulty to draw any conclusion about the evaluation of the OS because of the heterogeneity of the treatments used as second-line therapy.

**Figure 1:**
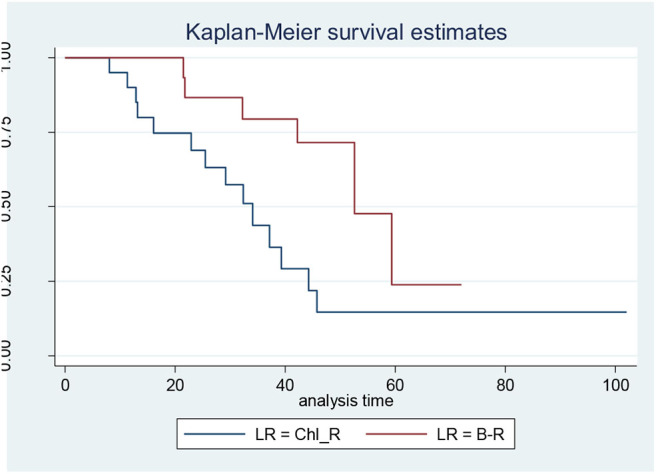
Progression-free survival in low-risk (LR) patients: bendamustine and rituximab LR vs. chlorambucil-R LR.

**Figure 2:**
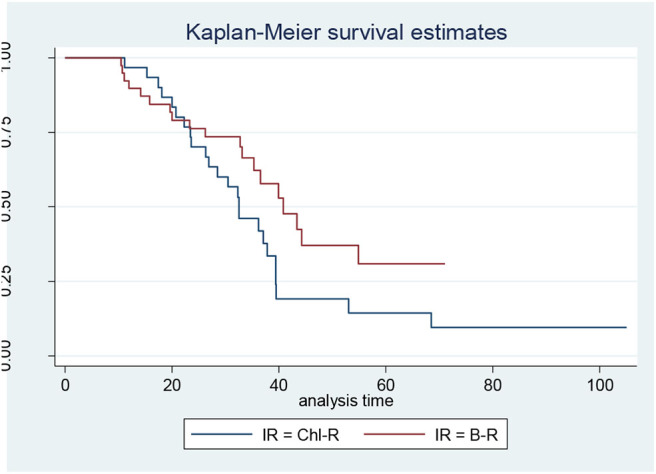
Progression-free survival in intermediate-risk (IR) patients: bendamustine and rituximab IR vs. chlorambucil-R IR.

When the CLL patients were grouped according to their biological characteristics in the IR and the LR subgroups, the two treatment arms did not show significantly different PFS, TTR, and OS rates. These data are discordant to those published by the GIMEMA study, in which the LR group showed better PFS and TTR when compared with IR (Figures 1, 2) (8).

As expected, the B-R group showed higher hematological toxicity compared to the Chl-R arm (*p* = 0.007). Grades III and IV hematological toxicity, especially neutropenic events, was higher in the B-R group; G-CSF was used in 23% of B-R patients and 6% of Chl-R patients, respectively. The B-R arm also presented significantly higher extra-hematological toxicity episodes than the Chl-R group (*p* = 0.008), especially for infective complications (24% in B-R and 11% in Chl-R); hospitalizations were necessary in 14 and 6% of CLL patients treated with B-R and Chl-R, respectively. Bendamustine and chlorambucil reduction was registered in 34 and 19% of patients, respectively. Dose reductions and toxicities were not related to age. These side effects were as expected for the CLL patients receiving chemoimmunotherapy (9, 11, 15).

## Conclusions

This retrospective study confirms the feasibility of B-R and Chl-R in elderly untreated CLL patients. The very elderly and unfit patients are usually treated with Chl-R, even if only B-R treatment did not negatively affect the OS rate. The Chl-R scheme allows achieving the same ORR, PFS, TTR, and OS when compared with B-R because of hematological and extra-hematological toxicities due to B, in which a greater dose reduction has been shown in comparison to Chl.

## Data Availability Statement

The datasets generated for this study are available on request to the corresponding author.

## Ethics Statement

The studies involving human participants were reviewed and approved by Ethics Committee of ‘Fondazione Policlinico Gemelli’ (Prot N 0035890/17 del 25/07/2017).

## Author Contributions

FA, II, and LL: conceptualization. FC and AF: formal analysis. FC, MD, SR, PF, EC, ML, DR, LD, AA, FP, GC, AT, MM, FM, and GD: data curation. FA and LL: original draft preparation, review and editing. II, FC, AA, GC, AT, MM, FM, and GD: supervision.

## Conflict of Interest

The authors declare that the research was conducted in the absence of any commercial or financial relationships that could be construed as a potential conflict of interest.
